# Molecular Evidence of Canine Distemper Virus Circulation in Red Foxes and Golden Jackals from Western Romania

**DOI:** 10.3390/microorganisms14081651

**Published:** 2026-07-29

**Authors:** Florin Vlad Gheorghe, Vlad Iorgoni, David Purec, Diana Maria Degi, Janos Degi, Cristian Zaha, Bogdan Alexandru Florea, Viorel Herman

**Affiliations:** 1 Department of Infectious Diseases and Preventive Medicine, University of Life Science “King Michael I” from Timisoara, 300645 Timisoara, Romania; florin.vlad@usvt.ro (F.V.G.); vlad.iorgoni@usvt.ro (V.I.); david.purec.fmv@usvt.ro (D.P.); viorelherman@usvt.ro (V.H.); 2 Faculty of Bioengineering of Animal Resources, University of Life Science “King Michael I” from Timisoara, 300645 Timisoara, Romania; degi.diana-maria.fbira@usvt.ro; 3 Department of Small Animal Surgery, University of Life Science “King Michael I” from Timisoara, 300645 Timisoara, Romania; cristian.zaha@usvt.ro; 4 Department of Internal Medicine, University of Life Science “King Michael I” from Timisoara, 300645 Timisoara, Romania; bogdan-alexandru.florea.fmv@usvt.ro

**Keywords:** canine distemper virus, wild canids, red fox, golden jackal, RT-qPCR, wildlife surveillance, Romania

## Abstract

Canine distemper virus (CDV) is a highly contagious multi-host pathogen that poses a significant threat to both domestic and wild carnivore populations. The increasing overlap between wildlife habitats, peri-urban environments, and domestic animal populations has raised concerns regarding the epidemiological role of wild canids in maintaining and disseminating CDV. This study investigated the occurrence of CDV infection among free-ranging wild canids from western Romania. Brain tissue samples collected from 64 free-ranging wild canids, including 38 red foxes (*Vulpes vulpes*) and 26 golden jackals (*Canis aureus*), were initially screened using a commercial immunochromatographic antigen detection assay. Positive samples were subsequently subjected to molecular confirmation by real-time reverse transcription polymerase chain reaction (RT-qPCR). CDV antigen was detected in 16 of the 64 investigated animals (antigen-positive proportion: 25.0%), including 12 red foxes (31.6%) and 4 golden jackals (15.4%). All antigen-positive samples were subsequently confirmed by RT-qPCR, demonstrating the presence of CDV RNA in the screened-positive subset. Since molecular testing was performed only on antigen-positive samples, these proportions should not be interpreted as molecular prevalence estimates. Viral RNA was confirmed in all antigen-positive samples by RT-qPCR, providing molecular evidence of CDV infection in opportunistically sampled wild canids. Although a higher antigen-positive proportion was observed in red foxes, no statistically significant association was identified between host species and CDV positivity (χ^2^ = 2.16, *p* = 0.142). These findings provide molecular confirmation of CDV infection in opportunistically collected rabies-negative wild canid carcasses from western Romania. Although the study demonstrates the presence of CDV in both investigated species, the sampling strategy does not allow estimation of population-level occurrence or broader epidemiological inference. Continuous molecular surveillance integrating wildlife, domestic animals, and environmental monitoring is warranted to improve understanding of CDV epidemiology and transmission dynamics in southeastern Europe within a One Health framework.

## 1. Introduction

Canine distemper virus (CDV) is a highly contagious member of the genus *Morbillivirus* (family *Paramyxoviridae*) that infects a wide range of domestic and wild carnivore species [1,2,3]. Owing to its broad host range and ability to cross species barriers, CDV remains an important pathogen affecting wildlife conservation and domestic animal health worldwide [1,4,5,6,7,8]. Outbreaks have been reported in numerous free-ranging carnivore populations, including both common and endangered species, highlighting the importance of continued surveillance in wildlife reservoirs [3,9,10,11].

In Europe, increasing ecological overlap between wild carnivores, domestic dogs, and human-modified environments has raised concerns regarding the circulation of CDV at the wildlife–domestic animal interface [8,12,13,14,15]. Among European wild canids, the red fox (*Vulpes vulpes*) and the golden jackal (*Canis aureus*) are of particular interest because of their wide distribution, ecological adaptability, and frequent occurrence in agricultural and peri-urban habitats. The recent expansion of golden jackal populations throughout southeastern and central Europe further emphasizes the need for continued surveillance of infectious diseases in free-ranging carnivores [16].

Despite the recognized importance of CDV in European wildlife, information regarding its occurrence in wild canids from Romania remains limited, particularly in the western part of the country, where wildlife habitats frequently overlap with rural settlements and domestic dog populations. Moreover, the epidemiological significance of CDV detection in free-ranging carnivores cannot be fully understood without systematic surveillance and complementary molecular characterization, including viral sequencing and comparative investigations involving sympatric domestic animals.

Therefore, the present study aimed to investigate CDV infection in opportunistically collected rabies-negative red fox and golden jackal carcasses from western Romania using a two-step diagnostic approach based on rapid antigen screening followed by molecular confirmation by real-time reverse transcription polymerase chain reaction (RT-qPCR). The study was designed to provide molecular confirmation of CDV infection in the investigated animals and to generate baseline surveillance data supporting future epidemiological and molecular investigations of CDV in wildlife from Romania.

## 2. Materials and Methods

### 2.1. Study Area and Sample Collection

The study was carried out in Timiș County, western Romania, a region characterized by a heterogeneous landscape comprising agricultural areas, forest habitats, wetlands, and peri-urban ecosystems that support diverse wildlife populations. Brain tissue samples were collected from free-ranging wild canids during the 2022–2023 surveillance period.

A total of 64 wild canid carcasses were included in the study, consisting of 38 red foxes (*Vulpes vulpes*) and 26 golden jackals (*Canis aureus*). The investigated animals were found dead on hunting grounds and roadside areas and were submitted to the Timiș County Sanitary Veterinary and Food Safety Directorate (DSVSA Timiș) as part of routine wildlife rabies surveillance activities conducted within the Romanian National Rabies Control and Surveillance Programme. Brain tissue samples used in the present study were obtained from animals that tested negative for rabies during routine diagnostic investigations and were subsequently made available for additional virological analyses.

For each carcass, the available epidemiological data included the collection date, sex, and locality of origin. All 64 wild canids originated from Timiș County, Romania, and were collected through the passive rabies surveillance program between 2022 and 2023. Collection sites included Bacova, Izvin, Făget, Herneacova, Stanciova, Jabăr, Coșava, Bucovăț, and Fibiș. Carcasses were submitted during February, March, April, and November. Sex was recorded for all animals (38 males and 26 females), while the estimated age, assessed by the submitting veterinarian, ranged from approximately 1 to 8 years. Information regarding carcass condition and the presumed cause of death was not consistently available and was therefore not included in the analyses.

Brain tissue samples obtained from rabies-negative wild canids were provided by the Timiș County Sanitary Veterinary and Food Safety Directorate (DSVSA Timiș) and transported under refrigerated conditions to the diagnostic laboratory of the Faculty of Veterinary Medicine, University of Life Sciences “King Mihai I” from Timisoara. The investigated animals consisted exclusively of red fox (*Vulpes vulpes*) and golden jackal (*Canis aureus*) carcasses found dead on hunting grounds or roadside areas within Timiș County, western Romania. The carcasses were submitted by the competent veterinary authorities through the Romanian National Rabies Surveillance Programme and included in the present study only after testing negative for rabies. No animals were intentionally captured, euthanized, or shot specifically for this study. Consequently, the study population represents a convenience sample of rabies-negative wild canids and should not be considered representative of the free-ranging wild canid populations in western Romania. Upon arrival, the samples were processed under appropriate biosafety conditions, handled as required, and stored at −20 °C until further laboratory analysis.

### 2.2. CDV Detection

Canine distemper virus (CDV) antigen detection was initially performed using a commercial immunochromatographic assay (Anigen Rapid CDV Ag Test Kit; BioNote Inc., Hwaseong, Republic of Korea) according to the manufacturer’s instructions. Brain tissue samples were homogenized in sterile phosphate-buffered saline prior to analysis. The commercial assay was used exclusively as an initial screening tool and was applied off-label to post-mortem brain homogenates from wild canids, as this specimen type has not been formally validated by the manufacturer.

All antigen-positive samples were subsequently subjected to molecular confirmation by real-time reverse transcription polymerase chain reaction (RT-qPCR), which served as the confirmatory diagnostic method. Viral RNA was extracted using the Direct-zol RNA MiniPrep Kit (Zymo Research, Irvine, CA, USA). RT-qPCR was performed using the Luna^®^ Universal One-Step RT-qPCR Kit (New England Biolabs, Ipswich, MA, USA), targeting a conserved region of the CDV genome with primers and probe previously described by Elia et al. [2]. The assay targeted an 87-bp fragment of the CDV genome using the following oligonucleotides: forward primer CDV-F (5′-AGCTAGTTTCATCTTAACTATCAAATT-3′), reverse primer CDV-R (5′-TTAACTCTCCAGAAAAACTCATGC-3′), and the TaqMan hydrolysis probe CDV-Pb (5′-FAM-ACCCAAGAGCCCGGATACATAGTTTCAATGC-TAMRA-3′).

### 2.3. Interpretation of RT-qPCR Results

RT-qPCR was used as a qualitative confirmatory assay. Amplification plots were interpreted using MyGo software (version 3.5.2). A sample was considered positive when a characteristic exponential amplification curve was observed together with a valid positive control, while no amplification was observed in the negative control. As the assay was used exclusively for qualitative confirmation according to the published protocol, no predefined Ct threshold was applied.

### 2.4. Statistical Analysis

Descriptive statistical analysis was performed to summarize the proportion of CDV antigen-positive animals among the investigated wild canid species. Antigen-positive proportions were expressed as percentages of positive samples relative to the total number of animals examined, together with their corresponding 95% confidence intervals calculated using the Wilson score method.

Associations between host species and CDV positivity were evaluated using Pearson’s chi-square test. Statistical significance was established at *p* < 0.05. All statistical analyses were conducted using IBM SPSS Statistics software (version 26.0; IBM Corp., Armonk, NY, USA).

### 2.5. Ethical Considerations

All samples originated from wild canid carcasses submitted through the Romanian National Rabies Surveillance Program. No animals were captured or euthanized specifically for this study. The protocol was approved by the Bioethics Committee of the Faculty of Veterinary Medicine, University of Life Sciences “King Mihai I” from Timisoara, Romania.

## 3. Results

### Antigen-Positive Proportion of CDV Among Investigated Wild Canids

A total of 64 brain tissue samples obtained from free-ranging wild canid carcasses were included in the study and analyzed for the presence of canine distemper virus (CDV). The study population comprised 38 red foxes (*Vulpes vulpes*) and 26 golden jackals (*Canis aureus*), all originating from Timiș County, Romania. Carcasses were collected through the passive rabies surveillance program between 2022 and 2023, during the months of February, March, April, and November, from nine localities (Bacova, Izvin, Făget, Herneacova, Stanciova, Jabăr, Coșava, Bucovăț, and Fibiș). Of the investigated animals, 38 (59.4%) were males, and 26 (40.6%) were females, while the estimated age, as assessed by the submitting veterinarian, ranged from approximately 1 to 8 years. The epidemiological characteristics of the investigated wild canids are summarized in Table 1.

Initial screening using the immunochromatographic assay identified CDV antigen in 16 of the 64 analyzed samples, corresponding to an overall antigen-positive proportion of 25.0%. Among the positive cases, 12 samples originated from red foxes and four from golden jackals. Species-specific antigen-positive proportions were 31.6% (12/38) in red foxes and 15.4% (4/26) in golden jackals. The corresponding 95% confidence intervals reflected the limited precision of these estimates due to the relatively small sample size.

Subsequent RT-qPCR analysis confirmed the presence of CDV RNA in all antigen-positive samples. Because molecular testing was performed exclusively on samples that tested positive by the immunochromatographic antigen assay, the reported proportions represent antigen-screening results with RT-qPCR confirmation rather than molecular prevalence estimates for the investigated wild canid population. A Pearson’s chi-square test showed no statistically significant association between host species and antigen-positivity (RT-qPCR confirmed), although red foxes showed a higher antigen-positive proportion than golden jackals (χ^2^ ≈ 2.16, *p* ≈ 0.142).

The antigen-screening results and corresponding 95% Wilson confidence intervals are summarized in Table 2.

A Pearson’s chi-square test was performed to evaluate the association between host species and CDV positivity. Although a higher proportion of CDV antigen-positive animals was observed in red foxes compared to golden jackals, the difference between the two species was not statistically significant (χ^2^ = 2.16, *p* = 0.142).

## 4. Discussion

The present study provides molecular confirmation of CDV in antigen-positive free-ranging wild canids from western Romania. Among the 64 rabies-negative brain tissue samples included in the study, 16 (25.0%) tested positive using the rapid antigen assay, and all positive samples were subsequently confirmed by RT-qPCR. Positive animals were identified in both species investigated, namely red foxes and golden jackals. However, because molecular testing was performed exclusively on antigen-positive samples, the reported findings represent antigen-positive animals subsequently confirmed by RT-qPCR rather than molecular prevalence estimates for the investigated wild canid population.

The detection of RT-qPCR-confirmed CDV infection in both species is consistent with previous reports describing CDV infection in free-ranging wild canids from Eastern and Southeastern Europe [8,16,17,18]. Nevertheless, the present study was based on opportunistically collected rabies-negative carcasses and included a relatively limited number of animals. Therefore, the observed antigen-positive proportions should not be interpreted as prevalence estimates or as evidence of biological differences between species. Although a higher antigen-positive proportion was observed in red foxes (31.58%) than in golden jackals (15.38%), this difference was not statistically significant. Consequently, within the limitations of the present study, no conclusions can be drawn regarding species-specific susceptibility, reservoir competence, maintenance mechanisms, or transmission pathways.

An important strength of the present study is the integration of molecular confirmation into the national passive rabies surveillance system. Existing wildlife surveillance programs may provide valuable opportunities for the opportunistic detection of additional pathogens without the need for dedicated wildlife sampling campaigns. The use of RT-qPCR to confirm all antigen-positive samples strengthened the diagnostic reliability of the findings, particularly because rapid immunochromatographic assays may be influenced by sample quality, viral load, and post-mortem changes [6,19]. However, the commercial antigen assay employed for the initial screening has not been formally validated for post-mortem brain tissue or wildlife species, and antigen-negative animals were not examined by RT-qPCR. Consequently, false-negative screening results cannot be excluded, and the diagnostic approach used in the present study should be regarded as antigen screening followed by molecular confirmation rather than a molecular survey of all investigated animals.

Several limitations should be considered when interpreting the present findings. The investigated animals originated exclusively from opportunistic rabies surveillance submissions and consisted of rabies-negative carcasses collected in Timiș County, western Romania. Consequently, the study population is subject to selection bias and cannot be considered representative of the free-ranging wild canid population. In addition, sequencing and phylogenetic analyses were not performed, preventing the characterization of circulating viral lineages or the assessment of possible epidemiological links with domestic dogs or neighboring countries. Furthermore, epidemiological information regarding carcass condition and the presumed cause of death was not consistently available and could therefore not be included in the analyses.

Overall, the present findings provide preliminary molecular evidence of CDV infection in red foxes and golden jackals opportunistically sampled through the passive rabies surveillance program in western Romania. They also highlight the value of passive rabies surveillance as a source of samples for the opportunistic detection of wildlife pathogens within a One Health framework. Future studies should incorporate systematic sampling, molecular testing of all collected specimens, viral sequencing, and comparative investigations involving both wildlife and domestic dogs to improve understanding of the molecular epidemiology and ecology of CDV in Romania [3,4,15,18].

## 5. Conclusions

This study provides molecular confirmation of canine distemper virus (CDV) infection in opportunistically collected rabies-negative red fox and golden jackal carcasses from western Romania. Because the investigated animals originated from passive rabies surveillance and RT-qPCR was performed only on antigen-positive samples, the findings should not be interpreted as estimates of population-level CDV occurrence. Instead, they provide preliminary molecular evidence supporting the value of opportunistic wildlife surveillance combined with molecular confirmation for detecting CDV infection in free-ranging wild canids.

CDV infection was identified in both species investigated, although the apparent antigen-positive proportion was higher in red foxes than in golden jackals. However, this difference was not statistically significant, and, given the limited sample size and opportunistic sampling strategy, no conclusions can be drawn regarding species-specific susceptibility, reservoir competence, or the epidemiological role of either species.

Overall, the present study contributes baseline surveillance data on CDV infection in wild canids from western Romania and highlights the need for larger, systematically designed investigations incorporating molecular testing of all collected samples, viral sequencing, and comparative surveillance of wildlife and domestic carnivores to improve understanding of CDV epidemiology in southeastern Europe.

## Figures and Tables

**Table 1:** Epidemiological characteristics of the investigated wild canids (*n* = 64).

Variable	Category	*n* (%)
Study period	2022–2023	–
Months of collection	February, March, April, November	–
County of origin	Timiș	64 (100)
Localities	Bacova, Izvin, Făget, Herneacova, Stanciova, Jabăr, Coșava, Bucovăț, Fibiș	–
Species	Red fox (*Vulpes vulpes*)	38 (59.4)
	Golden jackal (*Canis aureus*)	26 (40.6)
Sex	Male	38 (59.4)
	Female	26 (40.6)
Estimated age	Approximately 1–8 years	–

Carcasses were submitted through the passive rabies surveillance program. Collection date, sex, locality of origin, and county were available for all animals. Estimated age was provided by the submitting veterinarian. Information regarding carcass condition and the presumed cause of death was not consistently available and was therefore not included in the analyses.

**Table 2:** Antigen-screening results in red foxes and golden jackals with RT-qPCR confirmation of antigen-positive samples.

Species	Examined Animals (*n*)	Antigen-Positive Samples (*n*)	Antigen-Positive Proportion (%)	95% CI (Wilson)
Red fox	38	12	31.6	19.1–47.5
Golden jackal	26	4	15.4	6.2–33.5
Total	64	16	25.0	16.0–36.8

Only antigen-positive samples were subjected to RT-qPCR confirmation. Therefore, the reported proportions represent antigen-screening results rather than molecular prevalence estimates. Wilson 95% confidence intervals are shown.

## Data Availability

The data presented in this study are available on reasonable request from the corresponding author. The data is not publicly available due to ethical and institutional restrictions.

## Abbreviations

The following abbreviations are used in this manuscript:

CDV	Canine Distemper Virus
RT-qPCR	Real-Time Reverse Transcription Polymerase Chain Reaction
RNA	Ribonucleic Acid
PCR	Polymerase Chain Reaction
DSVSA	Sanitary Veterinary and Food Safety Directorate (Direcția Sanitară Veterinară și pentru Siguranța Alimentelor)
PBS	Phosphate-Buffered Saline
Ag	Antigen
χ^2^	Pearson’s Chi-Square Statistic
CDV-F	Forward Primer for Canine Distemper Virus Detection
CDV-R	Reverse Primer for Canine Distemper Virus Detection
CDV-Pb	Hydrolysis Probe for Canine Distemper Virus Detection
EU	European Union
CDV RNA	Canine Distemper Virus Ribonucleic Acid
RT	Reverse Transcription
*V. vulpes*	*Vulpes vulpes* (Red Fox)
*C. aureus*	*Canis aureus* (Golden Jackal)
